# The First Metal Complexes of 4,6-diamino-1-hydro-5-hydroxy-pyrimidine-2-thione: Preparation, Physical and Spectroscopic Studies, and Preliminary Antimicrobial Properties

**DOI:** 10.1155/2008/647873

**Published:** 2009-03-23

**Authors:** Sahar I. Mostafa, Constantina Papatriantafyllopoulou, Spyros P. Perlepes, Nick Hadjiliadis

**Affiliations:** ^1^Chemistry Department, Faculty of Science, Mansoura University, 35516 Mansoura, Egypt; ^2^Department of Chemistry, University of Patras, 265 04 Patras, Greece; ^3^Laboratory of Inorganic and General Chemistry, Department of Chemistry, University of Ioannina, 451 10 Ioannina, Greece

## Abstract

The new complexes [M_2_O_5_L_2_(H_2_O)_2_] · H_2_O (M = Mo, **1**; M = W, **2**), [RuL_2_(H_2_O)_2_] · H_2_O **(3)**, [ML_3_] · *x*H_2_O (M = Rh, *x* = 2, **4**; M = Ir, *x* = 1, **5**), [RhL_2_(PPh_3_)_2_](ClO_4_) · 2H_2_O **(6)**, [PdL_2_] · 2H_2_O **(7)**, [PdL(phen)]Cl · H_2_O **(8)**, [Re OL_2_(PPh_3_)]Cl **(9)** and [UO_2_L_2_] **(10)** are reported, where LH is 4,6-diamino-1-hydro-5-hydroxy-pyrimidine-2-thione. The complexes were characterized by elemental analyses, physical techniques (molar conductivity, room-temperature magnetic susceptibility), and spectroscopic (IR, Raman, UV/VIS/ligand field, NMR, mass) methods. The ligand L^−^ is in its thione form and behaves as a bidentate chelate with the deprotonated (hydroxyl) oxygen and the nitrogen of one amino group as donor atoms. Oxobridged dinuclear **(1, 2)** and various mononuclear **(3–10)** structures are assigned for the complexes in the solid state. The metal ion coordination geometries are octahedral **(1–6, 9, 10)** or square planar **(7, 8)**. The free ligand LH and complexes **1**, **4**, **7**, and **8** were assayed in vitro for antimicrobial activity against two bacterial and two fungal cultures.

## 1. INTRODUCTION

**Scheme 1:**
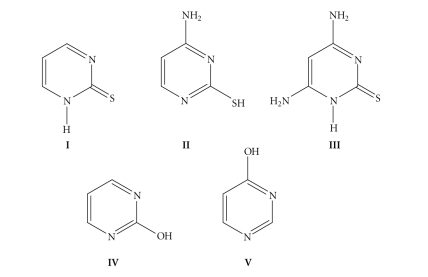
Structural formulae of pyrimidine-2-thione (**I**), and some of its amino (**II**, **III**) and hydroxy (**IV**, **V**) derivatives shown in one of their tautomeric forms.

2-Mercaptopyrimidine nucleotides have been detected in *Escherichia Coli* sRNA and yeast tRNA; it has been found that they inhibit the synthesis of tRNA, thus acting as antitumour and antithyroid agents [1]. A similar inhibitory effect has been observed for pyrimidine-2-thione (**I** in Scheme 1) and its derivatives, which also show pronounced in vitro bacteriostatic activity [1]. Metal complexes of pyrimidine-2-thione or its pyrimidine-2-thiol tautomeric form [1, 2] and its amino [2, 3] or hydroxy [4–6] derivatives have been prepared and studied (for representative ligands see Scheme 1). Such complexes exhibit rich structural chemistry, and interesting thermal, magnetic, sorptive, and biological properties. However, the coordination chemistry of ligands based on the 2-mercaptopyrimidine moiety and containing *both* hydroxy and amino substituents on the pyrimidine ring is completely unkown.

**Scheme 2:**
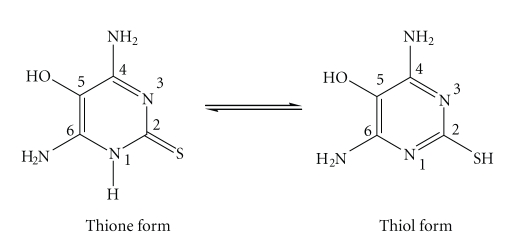
4,6-diamino-5-hydroxy-2-mercaptopyrimidine (LH).

We now describe here the preparation and characterization of the *first* metal complexes of 4,6-diamino-5-hydroxy-2-mercaptopyrimidine (LH, Scheme 2). We also report the antimicrobial activity of the free ligand and four representative complexes against two bacteria and two fungi. This work can be considered as a continuation of our interest on the coordination chemistry of derivatized pyrimidines [7].

## 2. EXPERIMENTS

All reagents were purchased from Merck and Alfa/Aesar. Na_2_[IrCl_6_] is commercially available. [PdCl_2_(phen)] was prepared by the reaction of K_2_[PdCl_4_] and 1,10-phenanthroline in H_2_O/EtOH. [ReOCl_3_(PPh_3_)_2_] was synthesized as previously reported [8]. DMSO used in conductivity measurements was dried over molecular sieves. The DMSO-d_6_ protons (NMR) were referenced using TMS. *Warning*: perchlorate salts are potentially explosive; such compounds should be used in small quantities and treated with utmost care at all times. Elemental analyses (C, H, N, S) were performed by the University of Ioannina (Greece) Microanalytical Unit with an EA 1108 Carlo-Erba analyzer. The water content of the complexes was also confirmed by TG/DTG measurements performed on a Shimadzu Thermogravimetric Analyzer TGA-50. IR spectra were recorded on a Matson 5000 FT-IR spectrometer with samples prepared as KBr pellets. Far-IR spectra were recorded on a Bruker IFS 113 v FT spectrometer with samples prepared as polyethylene pellets. FT Raman data were collected on a Bruker IFS 66 v interferometer with an FRA 106 Raman module, a CW Nd: YAG laser source, and a liquid nitrogen-cooled Ge detector. Solution electronic spectra were recorded using a Unicam UV_2−100_ spectrophotometer. Solid-state (diffuse reflectance, DRS) electronic spectra in the 300–800 nm range were recorded on a Varian Cary 3 spectrometer equipped with an integration sphere. ^1^H NMR studies were performed on a Varian Gemini WM-200 spectrometer. ^31^P{^1^H} NMR spectra were recorded with a Varian Mercury equipment [ref. 85% H_3_PO_4_ (ext.)]. Mass spectra were recorded on a Matson 5988 MS spectrometer. Conductivity measurements were carried out at room temperature on a YSI, model 32 conductivity bridge using 10^−3^ M solutions. Room temperature magnetic susceptibility measurements were performed using a Johnson Matthey magnetic balance standardized with HgCo(NCS)_4_; diamagnetic corrections were estimated using Pascal's constants.

### 2.1. Preparation of the complexes

**[Mo_2_O_5_L_2_(H_2_O)_2_] · H_2_O (1)**

An aqueous solution (5 cm^3^) of (NH_4_)_2_[MoO_4_] (0.24 g, 1.0 mmol) was added to a solution of LH (0.16 g, 1.0 mmol) in EtOH (25 cm^3^). The obtained slurry was heated and the resulting orange solution was refluxed for 4 hours, during which time an orange precipitate is formed. The solid was collected by filtration, washed with ethanol (2 cm^3^) and diethyl ether (2 × 5 cm^3^) and dried in vacuo. The yield was 35% (based on the metal). Elemental analytical calculation for C_8_H_16_N_8_O_10_S_2_Mo: C, 15.00; H, 2.50; N, 17.50; S, 10.00% found that C, 14.98; H, 2.82; N, 17.51; S, 9.87%; Λ_M_(DMSO): 3 S cm^2^ mol^−1^.

**[W_2_O_5_L_2_(H_2_O)_2_] · H_2_O (2)**

Using (NH_4_)_2_[WO_4_] and following exactly the same procedure as that described for complex **1**, a bright yellow material was isolated. The yield was 50% (based on the metal). Elemental analytical calculation for C_8_H_16_N_8_O_10_S_2_W: C, 11.77; H, 1.96; N, 13.73; S, 7.85% found that C, 11.62; H, 1.90; N, 13.77; S, 7.95%; Λ_M_(DMSO): 2 S cm^2^ mol^−1^.

**[RuL_2_(H_2_O)_2_] · H_2_O (3)**

Solid RuCl_3_ · 3H_2_O (0.12 g, 0.46 mmol) was added to a solution of NaO_2_CMe (0.62 g, 7.5 mmol) in water (30 cm^3^). Solid LH (0.24 g, 1.5 mmol) was then added and the resultant reaction mixture was refluxed for 12 hours. The deep brown solid formed was collected by filtration while the reaction mixture was hot, washed with hot water, and dried in vacuo. The yield was 30% (based on the metal). Elemental analytical calculation for C_8_H_16_N_8_O_5_S_2_Ru: C, 20.46; H, 3.41;N, 23.88; S,13.64% found that C, 20.32; H, 3.05; N, 23.57; S, 13.21%; Λ_M_(DMSO): 1 S cm^2^ mol^−1^.

**[RhL_3_] · 2H_2_O (4)**

Using RhCl_3_ · 3H_2_O and following the same procedure as that described for complex **3**, a reddish brown material was isolated. The yield was 60% (based on the metal). Elemental analytical calculation for C_12_H_19_N_12_O_5_S_3_Rh: C, 23.61; H, 3.12; N, 27.55; S, 15.74% found that C, 23.73; H, 3.11; N, 26.36; S, 14.98%; Λ_M_(DMSO): 6 S cm^2^ mol^−1^.

**[IrL_3_] · H_2_O (5)**

Using Na_2_[IrCl_6_] and following the same procedure as that described for complex **3**, a yellow solid was isolated. The yield was 25% (based on the metal). Elemental analytical calculation for C_12_H_17_N_12_O_4_S_3_Ir: C, 21.14; H, 2.50; N, 24.66; S, 14.09% found that C, 21.33; H, 2.64; N, 24.75; S, 13.85%; Λ_M_(DMSO): 5 S cm^2^ mol^−1^.

**[RhL_2_(PPh_3_)_2_](ClO_4_) · 2H_2_O (6)**

A hot ethanolic solution (20 cm^3^) of LH (0.25 g, 1.6 mmol) was added to a solution of RhCl_3_ · 3H_2_O (0.21 g, 0.8 mmol) in 6 M HClO_4_ (15 cm^3^). The resultant orange solution was refluxed for 4 hours and to this was added a solution of PPh_3_ (0.43 g, 1.6 mmol) in hot ethanol (15 cm^3^). The new solution was refluxed for a further 3 hours and filtered, and its volume decreased in vacuo to give a red-brown solid. The solid was collected by filtration, washed with hot water (2 x 2 mL) and hot ethanol (2 x 3 cm^3^), and dried in vacuo. The yield was 25% (based on the metal). Elemental analytical calculation for C_44_CIH_44_N_8_O_8_S_2_P_2_Rh: C, 49.05; H, 4.09; N, 10.41; S, 3.30% found that C, 48.79; H, 4.09; N, 10.44; S, 3.46%; Λ_M_(DMSO): 48 S cm^2^ mol^−1^.

**[PdL_2_] · 2H_2_O (7)**

To a stirred slurry of LH (0.16 g, 1.0 mmol) in methanol (15 cm^3^) was added an aqueous solution (15 cm^3^) of K_2_[PdCl_4_] (0.16 g, 0.5 mmol). The resulting suspension was stirred at 40°C for 60 hours and the brown solid formed was collected by filtration, washed with water (5 × 3 cm^3^) and cold methanol (2 × 5 cm^3^), and dried in air. The yield (based on the metal) was 50%. Elemental analytical calculation for C_8_H_14_N_8_O_4_S_2_Pd: C,21.03; H, 3.07; N, 24.54; S, 14.02% found that C, 21.23; H, 3.22; N, 24.85; S, 14.21%; Λ_M_(DMSO): 9 S cm^2^ mol^−1^.

**[PdL(phen)]Cl · H_2_O (8)**

To a stirred yellow slurry of [PdCl_2_(phen)] (0.18 g, 0.5 mmol) in a methanol/benzene solvent mixture (15 cm^3^, 3:2 v/v) was added a solution of KOH (0.055 g, 1.0 mmol) in methanol (15 cm^3^). Solid LH (0.08 g, 0.5 mmol) was added to the reaction mixture which soon dissolved. The solution was filtered and stirred for 48 hours at room temperature. During this time, a brown precipitate formed which was collected by filtration, washed with water (1 cm^3^) and methanol (2 × 3 cm^3^), and dried in air. The yield was 40% (based on the ligand). Elemental analytical calculation for C_8_H_14_N_8_O_4_S_2_Pd: C, 21.03; H, 3.07; N, 24.54; S, 14.02% found that C, 21.23; H, 3.22; N, 24.85; S, 14.21%; Λ_M_(DMSO): 77 S cm^2^ mol^−1^.

**[ReOL_2_(PPh_3_)]CL (9)**

To a stirred slurry of [ReOCl_3_(PPh_3_)_2_] (0.25 g, 0.2 mmol) in ethanol (30 cm^3^) was added solid LH (0.057 g, 0.4 mmol). The solid soon dissolved and stirred at 40°C for 5 hours. The brown solution deposited a brown microcrystalline solid which was collected by filtration, washed with ethanol (3 × 3 cm^3^), and dried in vacuo. The yield was 65% (based on the metal). Elemental analytical calculation for C_26_CIH_25_N_8_O_3_S_2_Re: C, 38.34; H, 3.07; N, 13.76; S, 7.86% found that C, 38.37; H, 3.11; N, 13.87; S, 7.98%; Λ_M_(DMSO): 46 S cm^2^ mol^−1^.

**[UO_2_L_2_] (10)**

Solid LH (0.08 g, 0.5 mmol) was added to a stirred solution of [UO_2_(NO_3_)_2_] · 6H_2_O (0.25 g, 0.5 mmol) in methanol (10 cm^3^). The solid soon dissolved. The resultant yellow solution was filtered and refluxed for 4 hours, during which time a red microcrystalline solid was precipitated. The product was collected by filtration, washed with methanol (5 cm^3^) and diethyl ether (2 × 5 cm^3^), and dried in vacuo. The yield was 55% (based on the metal). Elemental analytical calculation for C_8_H_10_N_8_O_4_S_2_U: C,16.44; H, 1.71; N, 19.18; S, 10.96% found that C, 16.35; H, 2.02; N, 18.98; S, 10.86%; Λ_M_(DMSO): 11 S cm^2^ mol^−1^.

### 2.2. Antimicrobial activity

The bacterial strains (*S. aureus* and *P. aeruginosa*) were grown in Nutrient agar slants and the fungal strains (*A. niger* and *C. albicans*) were grown in Sabouraud dextrose agar slants. The viable bacterial cells were swabbed onto Nutrient agar plates, while the fungal spores onto Sabouraud dextrose agar plates. The free ligand and complexes **1**, **4**, **7** were dissolved in DMSO, while complex **8** was dissolved in H_2_O with 10, 20, 50, and 100 mg/mL concentrations. The blank was DMSO in saline buffer. The bacterial and fungal plates were incubated for 36 and 72 hours, respectively, and the activity of the compounds was estimated by measuring the diameter of the inhibition zone (the affected zone by the compounds) around the respective zone (the normal place in the agar). The incubation temperature was 27 ± 0.5°C.

## 3. RESULTS AND DISCUSSION

### 3.1. Synthetic comments and physical characterization

The preparative reactions for selected complexes can be represented by the stoichiometric equations (1)–(7); no attempts were made to optimize the yields,

(1) $$\begin{array}{ll} & {2({\text{NH}}_{4})}_{2}[{\text{MO}}_{4}]+2\text{LH}\\ & \overset{\text{EtOH}/{\text{H}}_{2}\text{O}}{\underset{\text{T}}{\to }}[{\text{M}}_{2}{\text{O}}_{5}{\text{L}}_{2}{\left({\text{H}}_{2}\text{O}\right)}_{2}]\\ & \;\;\;\;\;\text{ M = Mo }(1)\text{, W}(2), \end{array}+4{\text{NH}}_{3}+{\text{H}}_{2}\text{O}$$

(2) $$\begin{array}{l} \begin{array}{ll} & \text{RhC}{\text{l}}_{3}\cdot 3{\text{H}}_{2}\text{O}+3{\text{LH+3NaO}}_{2}\text{CMe}\\ & \overset{{\text{H}}_{2}\text{O}}{\underset{\text{T}}{\to }}[{\text{RhL}}_{3}](4)+3\text{NaCl}+3{\text{MeCO}}_{2}\text{H}+3{\text{H}}_{2}\text{O,} \end{array} \end{array}$$

(3) $$\begin{array}{ll} & \text{RhC}{\text{l}}_{3}\cdot 3{\text{H}}_{2}\text{O}+2{\text{LH+2PPh}}_{2}+{\text{HCIO}}_{4}\\ & \overset{\text{EtOH}/{\text{H}}_{2}\text{O}}{\underset{\text{T}}{\to }}\left[{\text{RhL}}_{2}{\left({\text{PPh}}_{3}\right)}_{2}\right]({\text{ClO}}_{4})(6)+3\text{HCl}+3{\text{H}}_{2}\text{O,} \end{array}$$

(4) $$\begin{array}{ll} & {\text{K}}_{2}[{\text{PdCl}}_{4}]+2\text{LH}\\ & \overset{\;\text{MeOH/}{\text{H}}_{2}\text{O}}{\to }[{\text{PdL}}_{2}](7)+2\text{K}\;\text{Cl}+2\text{H}\;\text{Cl,} \end{array}$$

(5) $$\begin{array}{ll} & [{\text{PdCl}}_{2}(\text{phen})]+\text{LH}+\text{KOH}\\ & \overset{\text{MeOH}/\text{benzene}}{\to }\left[\text{PdL}(\text{phen})\right]\text{Cl}(8)+\text{KCl}+{\text{H}}_{2}\text{O,} \end{array}$$

(6) $$\begin{array}{ll} & \left[{\text{Re}{\text{OCl}}_{3}({\text{PPh}}_{3})}_{2}\right]+2\text{LH}\\ & \overset{\text{EtOH}}{\to }[{\text{Re}{\text{Ol}}_{2}({\text{PPh}}_{3})}_{2}]\text{Cl}(9)+2\text{HCl}+{\text{PPh}}_{3}, \end{array}\;$$

(7) $$\begin{array}{ll} & \left[{\text{UO}}_{2}{\left({\text{NO}}_{3}\right)}_{2}\right]\cdot 6{\text{H}}_{2}\text{O}+2\text{LH}\\ & \overset{\text{MeOH}}{\underset{\text{T}}{\to }}\left[{\text{UO}}_{2}{\text{L}}_{2}\right](10)+2{\text{HNO}}_{3}+6{\text{H}}_{2}\text{O.} \end{array}$$

The metal is reduced (Ru^III^ → Ru^II^, Ir^IV^ → Ir^III^) during the preparation of complexes **3** and **5** although the reactions are performed in air. The redox reaction may be facilitated by the reducing character of LH, the products from the oxidation of the ligand remaining in the solution. Thus, LH possibly plays two roles in the reactions, that is, the role of the ligand and that of the reducing agent. It is well known that Ru(III) can undergo reduction reactions and that the [Ir^IV^Cl_6_]^2−^ ion is a convenient one-electron oxidant [9]. The use of a base (KOH) in the preparation of **8** is necessary to obtain the complex in pure form; otherwise, the produced aqueous HCl decomposes the compound.

Complexes **1**–**5**, **7** and **10** are nonelectrolytes in DMSO [10]. Complexes **7** and **10** exhibit slightly increased molar conductivity values in DMSO. Since DMSO is a good donor solvent, this may be due to the partial displacement of one L^−^ ligand by two DMSO molecules. Assuming an equilibrium between the neutral and the resulting cationic complex, this displacement changes the electrolyte type of the compound explaining the increased Λ_M_ value [10]. From the molar conductivities in DMSO (complexes **6** and **9**) and DMF (complex **8**), it is concluded that compounds **6**, **8**, and **9** behave as 1 : 1 electrolytes, supporting their ionic formulation [10]. All the complexes are diamagnetic, as expected [9]. It should be mentioned at this point that the *π* bonding in the {Re^V^ = O}^3+^ unit of **9** causes sufficient splitting of the *t*_2*g*_ (in *O_*h*_*) set (5d_*xz*_, 5d_*yz*_ ≫ 5d_*xy*_) that diamagnetism occurs through the configuration (5d_*xy*_)^2^.

Complexes **1**–**10** are microcrystalline or powder-like, stable in the normal laboratory atmosphere, and soluble only in DMF and DMSO. We had hoped to structurally characterized one or two complexes by single-crystal X-ray crystallography (working mainly with DMF or DMF/MeCN), but were thwarted on numerous occasions by twinning problems or lack of single crystals. Thus, the characterization of the complexes is based on spectroscopic methods.

### 3.2. Electronic spectra

The band at 335 nm in the DRS spectrum of **1** is assigned to an O^2−^ → Mo^VI^ p–d LMCT transition and is characteristic of the {MoO_2_}^2+^ moiety [11] in octahedral complexes. The transition appears at 337 nm as a shoulder in solution (DMSO). The DRS spectrum of **3** is indicative of its low-spin octahedral structure. The ground term is ^1^ *A*_1*g*_ and the two spin-allowed transitions to ^1^ *T*_1*g*_ and ^1^ *T*_2*g*_ are observed at 565 and 420 nm, respectively [12]; the corresponding bands in DMSO are at 560 and 430 nm. The DRS spectra of the Rh(III) complexes **4** and **6** both exhibit bands at ~470 and ~380 nm; the spectra resemble those of other six-coordinate Rh(III) compounds and the bands are assigned as transitions from the ^1^ *A*_1*g*_ ground state to the ^1^ *T*_1*g*_ and ^1^ *T*_2*g*_ upper states in octahedral symmetry in decreasing order of wavelength [12]. The lower wavelength band may also have a charge-transfer character. Both complexes exhibit an additional band in the blue region of the spectrum (~520 nm) which is responsible for their red-brown colors; a possible origin of this band is the singlet-triplet, spin-forbidden transition ^1^ *A*_1*g*_ → ^3^ *T*_2*g*_ [12]. The spectrum of the Ir(III) complex **5** shows two bands at 380 and 335 nm, which have a similar interpretation; the ^1^ *A*_1*g*_ → ^3^ *T*_2*g*_ transition is not observed. A weak shoulder in the spectrum of **9** is assigned to the ^3^ *T*_1*g*_(*F*) → ^3^ *T*_2*g*_ transition in a d^2^ octahedral environment, while an intense band at 375 nm most probably has an LMCT origin [12]. The ligand-field spectra of **7** and **8** are typical of a square planar environment around pd ^II^ with a mixed N,O-ligation; the bands at 480, 375, and 330 nm are assigned [12] to the ^1^ *A*_1*g*_ → ^1^ *A*_2*g*_, ^1^ *A*_1*g*_ → ^1^ *E*_*g*_, and ^1^ *A*_1*g*_ → ^1^ *B*_1*g*_ transitions, respectively, under *D*_4*h*_ symmetry. The spectra in DMSO exhibit only two bands at 480 and 330 nm.

### 3.3. NMR studies

Diagnostic ^1^H NMR assignments (in DMSO-d_6_) for representative complexes are presented in Table 1. The study was based on comparison with the data obtained for diamagnetic complexes with similar ligands [7, 13, 14]. In all the spectra studied, the integration ratio of the signals is consistent with the assignments.

The spectrum of LH exhibits two singlets at *δ* 6.07 and 6.18 assigned to the –N(4)H_2_/–N(6)H_2_ (for the numbering scheme see Scheme 2) amino hydrogens, respectively, and two relatively broad singlets at *δ* 7.43 and 9.13 due to the amide and hydroxyl protons –N(1)H– and –O(5)H, respectively. The appearance of these four peaks is consistent with the exclusive presence of the thione form of LH (Scheme 2) in solution. The proton of the hydroxyl group is not observed in the spectra of the complexes confirming its deprotonation and coordination to the metal ions. In the spectra of **1**, **3**, **4**, and **6**–**8**, the –N(1)H– signal undergoes a marginal shift to indicate the noninvolvement of this group in coordination; a relatively large downfield shift would be expected if coordination had occurred. In the same spectra, two signals appear for –NH_2_ protons, as expected. The most pronounced variation in chemical shift is the downfield shift of one signal. Since more specific assignments of these two signals seem impossible, it is difficult to conclude which amino nitrogen is coordinated. NMR evidence for the presence of thione –thiol tautomerism in the metal complexes in solution was not found.

The ^1^H NMR spectrum of **4** confirms that the three N,O-bidentate (vide infra) ligands are equivalent (*C*_3_ symmetry), and, therefore, the complex has the *fac* stereochemistry [15].

The spectrum of **8** is indicative of the presence of one solution species containing coordinated phen, consisting of four resonances [16]. Assignments are as follows (the numbers in parentheses are the positions of the protons in the classical numbering scheme of 1,10-phenanthroline; s = singlet, dd=doublet of doublets): 9.15 dd(2H; 2,9), 8.53 dd(2H; 4,7), 8.00s(2H; 5,6), and 7.81q(2H; 3,8). Considerable downfield coordination shifts, Δ*δ*(H_*l*_)[Δ*δ*(H_*l*_) = *δ*(H_*l*_)_complex_ − *δ*(H_*l*_)_free phen_], are observed for all resonances, their values being 0.16, 0.27, 0.31, and 0.19 for the protons of the positions (2,9), (4,7), (5,6), and (3,8), respectively. These shifts are characteristic of coordinated phen [16].

The ^31^P{^1^H} NMR spectrum of the Re(V) complex **9** in DMSO-d_6_ consists of a sharp singlet at *δ* − 16.89, a value which is typical for PPh_3_-containing oxorhenium(V) species [17].

### 3.4. Vibrational spectra

Tentative assignments of selected IR ligand bands for complexes **1**–**10** and free LH are listed in Table 2. The assignments have been given by studying literature reports [3, 13, 14], comparing the spectrum of LH with the spectra of the complexes and by performing deuterium isotopic substitution experiments in few cases. As a general remark, we must emphasize that some stretching and deformation modes are coupled, so that the proposed assignments should be regarded as approximate descriptions of the vibrations.

In the *v*(OH)_water_ region, the spectra of complexes **1**–**3** show one medium-intensity band at ~3420 cm^−1^ attributed to the presence of coordinated water [13]. The same spectra exhibit, in addition to the relatively sharp band of coordinated water, a weaker broad continuous absorption covering the 3400–3200 cm^−1^ region; this is apparently due to the simultaneous presence of crystal and coordinated water in these complexes [14]. In the spectra of **4**–**8**, a medium broad absorption indicates the presence of exclusively crystal (lattice) water.

The absence of an IR or Raman band at ~2600 cm^−1^ in the spectrum of free LH suggests that the ligand exists in its thione form (see Scheme 2) [18]. This is corroborated by the appearance of the medium *v*(C=S) band at 1177 cm^−1^ (this vibration appears as a strong peak at 1160 cm^−1^ in the Raman spectrum) and the strong IR *v*(N–H) band at 2970 cm^−1^ (this vibration appears as a medium peak at ~3000 cm^−1^ in the Raman spectrum); the broadness and low frequency of the latter IR band are both indicative of the involvement of the –NH– group in strong hydrogen bonding.

The medium IR band at 3305 cm^−1^ in the spectrum of free LH is assigned to the *v*(OH) vibration. This band does not appear in the spectra of the complexes indicating deprotonation of the –OH group and suggesting coordination of the resulting, negatively charged oxygen atom. The absence of large systematic shifts of the *δ*(N–H), *δ*(NH), *v*(C=N), *v*(C_2_– N_1_)/*v*(C_2_– N_3_), and *v* (C=S) bands in the spectra of the complexes implies that there is no interaction between the ring nitrogen atoms or the exocyclic sulfur atom and the metal ions. The *v*_as_(NH_2_) and *v*_s_(NH_2_) bands are doubled in the spectra of the complexes. One band for each mode appears at almost the same wavenumber compared with the corresponding band in the spectrum of free LH, whereas the other band of each pair is significantly shifted to lower wavenumbers. This fact is a strong evidence for the presence of one coordinated and one “free” (i.e., uncoordinated) amino group per L^−^ in the complexes [7].

The presence of coordinated PPh_3_ groups in **6** and **9** is manifested by the strong IR bands at ~1100 and ~750 cm^−1^, attributed to the *v*(P–C) and *δ*(CCH) vibrations, respectively [17]; the former band overlaps with the ClO_4_^−^ stetching vibration in the spectrum of the Rh(III) complex **6**. In the spectrum of **8**, the bands at 1627, 1591, 1510, 1485, and 1423 cm^−1^ are due to the phen stretching vibrations [16]; these bands are at higher wavenumbers compared with the free phen indicating chelation. The bands at 854, 841, 743, and 725 cm^−1^ are assigned to the *γ*(CH) vibrations of the coordinated phen [16].

The vibrational spectra of the inorganic “parts” of complexes **1**, **2**, **6**, **9,** and **10** are also diagnostic. The IR spectrum of **6** exhibits a strong band at ~1100 and a medium band at 624 cm^−1^ due to the *v*_3_(*F*_2_) and *v*_4_(*F*_2_) modes of the uncoordinated *T*_d_ClO_4_^−^ ion [19], respectively, the former having also *v*(P–C) character [17]. In the 1000–750 cm^−1^ region, the spectra of **1** and **2** show bands characteristic of the *cis*-MO_2_^2+^ units and the {O_2_M–O–MO_2_}^2+^ core (M=Mo, W) [20, 21]. The IR bands at 930 and 912 cm^−1^ in **1** are assigned to the *v*_s_(MoO_2_) and *v*_as_(MoO_2_) modes, respectively [19, 20]; the corresponding Raman bands appear at 910 and 896 cm^−1^. As expected [19], the symmetric mode is weak in the IR spectrum and strong in the Raman spectrum, while the opposite applies for the asymmetric mode. The appearance of two stretching bands is indicative of the *cis* configuration [19]. The strong IR band at 745 cm^−1^ is assigned to the *v*_as_(Mo–O–Mo) mode [20], indicating the presence of a *μ*-O^2−^ group. The *v*_s_(WO_2_), *v*_as_(WO_2_), and *v*_as_(W–O–W) bands appear at 945, 922, and 755 cm^−1^, respectively, in the IR spectrum of complex **2** [19, 21]; the *v*_s_(WO_2_) and *v*_as_(WO_2_) Raman bands are at 940 and 917 cm^−1^, respectively. The *v*_s_(WO_2_) and *v*_as_(WO_2_) modes are at higher wavenumbers when compared to those of the analogous Mo(VI) complex **1**, suggesting [21] that the *cis*-WO_2_^2+^ group has some “triple” bond character [21]. In the spectra of **9**, the band attributed to **ν** (Re=O) appears at 956 (IR) and 968 (Raman) cm^−1^ [17, 19]. The IR spectrum of the uranyl complex **10** exhibits only one U=O stretching band, that is, *v*_as_(UO_2_), at 940 cm^−1^ (not observed in the Raman spectrum) indicating its linear transdioxo configuration [19]. The *v*_s_(UO_2_) mode appears as a strong Raman peak at 905 cm^−1^, and, as expected, the corresponding IR band is very weak. The bands at 345 and 298 cm^−1^ in the far-IR spectrum of **7** are assigned to the *v*(Pd–NH_2_) and *v*(Pd-O) vibrations, respectively. The appearance of one band for each mode (*B*_3*u*_ and *B*_2*u*_ under *D*_2*h*_) is consistent with a trans structure [19].

### 3.5. Antimicrobial activity studies

The free ligand LH and its complexes **1**, **4**, **7**, and **8** were assayed in vitro for antimicrobial activity against two bacterial (*S. aureus* and *P. aeruginosa*) and two fungal (*A. niger* and *C. albicans*) cultures. The hot plate diffusion method was adopted for the activity measurements [22]. Results are listed in Tables 3 and 4.

In general, the Pd(II) complexes **7** and **8** were found to have higher efficacy than **1**, **4**, and LH at the measured concentrations. The water-soluble complex **8** is the most active against the pathogens studied. It is remarkable that the antifungal activity of **8** is comparable with, or even better than, the activity of the antifungal drug nystatin, and this may be due to the simultaneous presence of phen and L^−^ in the complex. The activity of the Pd(II) complexes **7** and **8** is tentatively attributed to their inhibition of the DNA replication (by interacting with enzyme prosthetic groups and altering the microbial metabolism) and their ability to form hydrogen bonds with the cell wall and cell constituents [23]. The weaker activity of **4** is noteworthy; the reason for this is not clear.

## 4. CONCLUSIONS

**Scheme 3:**
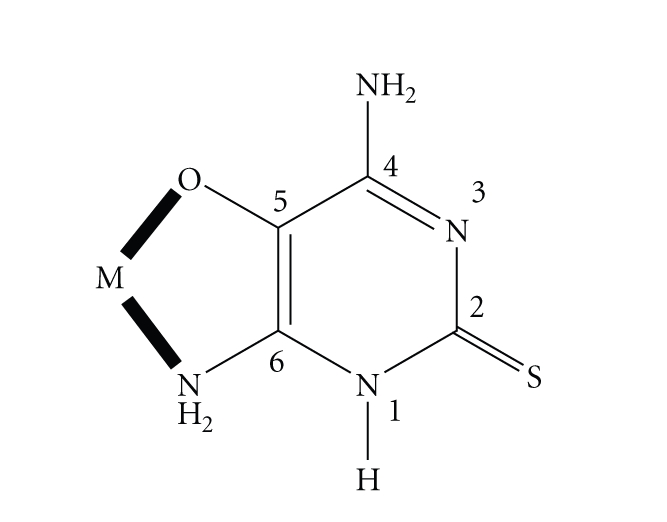
The proposed coordination mode of the anionic ligand L^−^ in complexes **1**–**10**; M=metal ion.

The M/LH general reaction system fulfilled its promise as a source of interesting complexes. From the overall evidence presented before, it seems that the ligand L^−^ behaves as a bidentate chelate in all the prepared complexes with the deprotonated oxygen and most probably the amino nitrogen of the position 6 of the pyrimidine ring being the donor atoms, see Scheme 3. However, the participation of the amino nitrogen of the position 4 of the ring cannot be ruled out. The nonparticipation of the sulfur atom in coordination in complexes **7** and **8** may be seen as unusual given the soft character of Pd(II) in the context of the HSAB concept.

**Figure 1:**
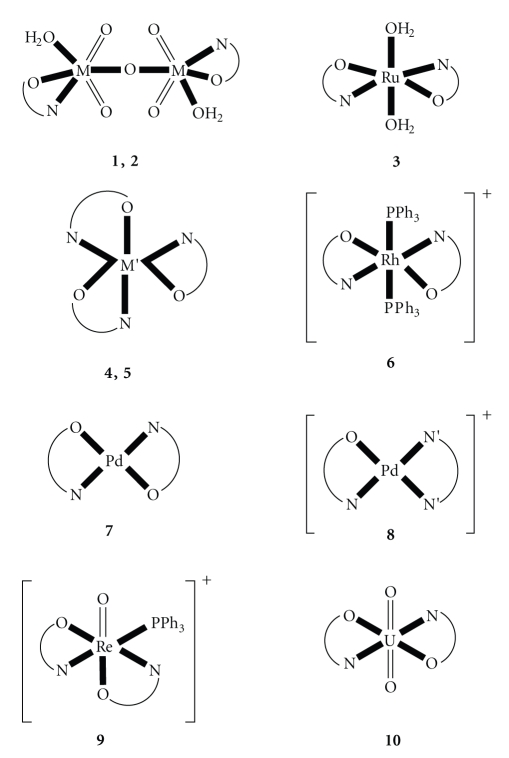
Schematic structures proposed for the neutral complexes **1**–**5**, **7**, **10** and for the cations of complexes **6**, **8**, **9**. Lattice H_2_O molecules and counterions have been omitted for clarity. However, $\overset{⌢}{\text{N}\;\text{O}}$ and $\overset{⌢}{{\text{N}}^{\prime }\;{\text{N}}^{\prime }}$ represent the ligands L^−^ and phen, respectively. The N and O donor atoms of L^−^ are the amino nitrogen of the position 6 (most probably) and the deprotonated oxygen of the position 5 of the pyrimidine ring; M=Mo, W; M′ = Rh, Ir.

The chelate effect (a stable chelating ring with the participation of the sulfur atom cannot be formed due to the geometry of L^−^) seems to govern the thermodynamic stability of these complexes. The proposed gross schematic structures for **1**–**10** are shown in Figure 1. Due to the fact that single-crystal, X-ray crystallographic studies are not available, few structural features (e.g., the symmetric structures of **1**–**3**, **6**, **7**, and **10**) are tentative. The metal ions adopt octahedral (**1**–**6**, **9**, **10**) or square planar (**7**, **8**) stereochemistries.

Finally, complexes **1**, **4**, **7**, and **8** are new welcome additions in the growing family of metal complexes with antimicrobial activity.

The results described in this report represent the initial study of the coordination chemistry of LH and the biological activity of its complexes. Further studies with 3d-metal ions are in progress.

## Figures and Tables

**Table 1:** Diagnostic^1^H NMR (*δ*, ppm)^a^ spectral data for LH and the representative complexes **1**, **3**, **4**, and **6**–**8** in DMSO-d_6_.

Compound	–N(1)H–	–N(4)H_2_/–N(6)H_2_	–O(5)H
LH	7.43	6.07, 6.18	9.13
**1**	7.57	6.18, 6.55	—
**3**	7.49	6.14, 6.47	—
**4**	7.44	6.20, 6.49	—
**6**	^b^	6.11, 6.47	—
**7**	7.47	6.17, 6.48	—
**8**	7.39	6.08, 6.46	—

^a^The spectra were run 10–15 min after dissolution. ^b^Obscured by the signals of the aromatic protons.

**Table 2:** Diagnostic ligand IR bands (cm^−1^) for LH and complexes **1**–**10**.

Compound	*v*_as_(NH_2_)^a^	*v*_s_(NH_2_)	*v*(N–H)	*v*(C=N), *v*(C=C)	*v*(C_2_– N_1_), *v*(C_2_– N_3_)	*δ*(NH)	*v*(C=S)
LH	3390	3185	2970	1652	1652	1455	1177
**1**	3377, 3330	3180, 3156	3005	1631	1553	1460	1155
**2**	3388, 3356	3188, 3160	3000	1633	1556	1465	1170
**3**	3399, 3343	3180, 3079	3010	1632	1558	1464	1157
**4**	3400, 3320	3160, 3105	3005	1642	1560	1460	1170
**5**	3387, 3320	3166, 3110	3010	1635	1556	1455	1177
**6**	3380, 3357	3170, 3095	3015	1641	1552^b^	1443^b^	1176
**7**	3395, 3357	3164, 3098	3010	1646	1550	1446	1170
**8**	3397, 3360	3165, 3100	3015	1640	1553	1450	1176
**9**	3400, 3355	3155, 3105	3012	1639	1555^b^	1460^b^	1170
**10**	3405, 3324	3180, 3100	3010	1641	1558	1462	1164

^a^Overlapping with the *v*(OH)_water_ band in the spectra of **1**–**8**. ^b^Overlapping with phenyl stretching vibrations of the coordinated PPh_3_ ligands.

**Table 3:** Diameters (mm) of growth inhibitions zones for the antibacterial activity of LH and complexes **1**, **4**, **7**, and **8**.

Compound	*S. aureus *(mg/cm^3^)^a^				*P. aeruginosa *(mg/cm^3^)^a^			
	10	20	50	100	10	20	50	100
LH	5	11	28	58	4	10	30	55
**8**	13	22	47	99	10	17	41	83
**7**	10	17	42	86	9	14	39	77
**4**	8	13	29	49	7	13	30	44
**1**	10	19	43	80	7	13	32	61

^a^mg/cm^3^ represents the concentration of the reagent in the gel.

**Table 4:** Diameters (mm) of growth inhibition zones for the antifungal activity of LH and complexes **1**, **4**, **7**, and **8**.

Compound	*A. niger *(mg/cm^3^)^a^				*C. albicans *(mg/cm^3^)^a^			
	10	20	50	100	10	20	50	100
LH	5	12	23	44	6	9	34	55
**8**	15	21	43	91	13	19	44	89
**7**	10	17	40	79	9	16	38	68
**4**	0	0	0	0	6	11	21	37
**1**	6	15	31	63	7	16	33	68
Nystatin^b^	17	19	36	65	20	26	42	79

^a^mg/cm^3^ represents the concentration of the reagent in the gel. ^b^A currently prescribed antifungal drug.
